# The European IPF registry (eurIPFreg): baseline characteristics and survival of patients with idiopathic pulmonary fibrosis

**DOI:** 10.1186/s12931-018-0845-5

**Published:** 2018-07-28

**Authors:** Andreas Guenther, Ekaterina Krauss, Silke Tello, Jasmin Wagner, Bettina Paul, Stefan Kuhn, Olga Maurer, Sabine Heinemann, Ulrich Costabel, María Asunción Nieto Barbero, Veronika Müller, Philippe Bonniaud, Carlo Vancheri, Athol Wells, Martina Vasakova, Alberto Pesci, Matteo Sofia, Walter Klepetko, Werner Seeger, Fotios Drakopanagiotakis, Bruno Crestani

**Affiliations:** 1 European IPF Registry & Biobank (eurIPFreg/bank), Giessen, Germany; 2 Universities of Giessen and Marburg Lung Center (UGMLC), Member of the German Center for Lung Research (DZL), Giessen, Germany; 3 Excellence Cluster Cardiopulmonary System (ECCPS), Justus-Liebig University Giessen and Johann Wolfgang Goethe University Frankfurt, Frankfurt, Germany; 4 AGAPLESION Lung Clinic Waldhof-Elgershausen, Greifenstein, Germany; 5 Reference Center for Rare Pulmonary Diseases, Centre Hospitalier Universitaire Dijon-Bourgogne, INSERMU1231, Université Bourgogne/Franche-Comté, Dijon, France; 6 Department of Clinical and Molecular Biomedicine, Università degli Studi di Catania, Catania, Italy; 7 Interstitial Lung Disease Unit, Royal Brompton Hospital, London, UK; 8 Ruhrlandklinik, University Hospital, Essen, Germany; 9 Hospital Clinico San Carlos, Madrid, Spain; 10 Department of Pulmonology, Semmelweis University, Budapest, Hungary; 11 First Faculty of Medicine and Thomayer Hospital, Prague, Czech Republic; 12 Ospedale San Gerardo, Monza, Italy; 13 Università degli Studi di Napoli Federico II, Naples, Italy; 14 Department of Thoracic Surgery, Vienna University Hospital, Vienna, Austria; 15 Competence Center for Rare Pulmonary Diseases, Hopital Bichat, Paris, France; 16 Clinical Research Unit “Pulmonary Fibrosis”, University of Giessen and Marburg Lung Center (UGMLC), German Center for Lung Research (DZL), European IPF Registry (eurIPFreg), Klinikstrasse 36, 35392 Giessen, Germany

**Keywords:** Idiopathic pulmonary fibrosis (IPF), European registry for idiopathic pulmonary fibrosis (eurIPFreg), Interstitial lung diseases (ILD)

## Abstract

**Background:**

Since 2009, IPF patients across Europe are recruited into the eurIPFreg, providing epidemiological data and biomaterials for translational research.

**Methods:**

The registry data are based on patient and physician baseline and follow-up questionnaires, comprising 1700 parameters. The mid- to long-term objectives of the registry are to provide clues for a better understanding of IPF phenotype sub-clusters, triggering factors and aggravating conditions, regional and environmental characteristics, and of disease behavior and management.

**Results:**

This paper describes baseline data of 525 IPF subjects recruited from 11/2009 until 10/2016. IPF patients had a mean age of 68.1 years, and seeked medical advice due to insidious dyspnea (90.1%), fatigue (69.2%), and dry coughing (53.2%). A surgical lung biopsy was performed in 32% in 2009, but in only 8% of the cases in 2016, possibly due to increased numbers of cryobiopsy. At the time of inclusion in the eurIPFreg, FVC was 68.4% ± 22.6% of predicted value, DLco ranged at 42.1% ± 17.8% of predicted value (mean value ± SD). Signs of pulmonary hypertension were found in 16.8%. Steroids, immunosuppressants and N-Acetylcysteine declined since 2009, and were replaced by antifibrotics, under which patients showed improved survival (*p* = 0.001).

**Conclusions:**

Our data provide important insights into baseline characteristics, diagnostic and management changes as well as outcome data in European IPF patients over time.

**Trial registration:**

The eurIPFreg and eurIPFbank are listed in ClinicalTrials.gov(NCT02951416).

## Background

Idiopathic pulmonary fibrosis (IPF) is a chronic, progressive, and usually fatal fibrotic disease of the lung that typically affects elder patients beyond 60 years of age. The overwhelming fibrotic processes and the distortion of the lung’s ultrastructure result in a progressive loss of pulmonary compliance and a decline in gas exchange properties [1]. The prevalence of the disease has been estimated at 2–29 per 100 000 persons and its incidence is about 3–9 per 100,000 persons per year [2, 3]. The average survival from the time of diagnosis is estimated as three to five years [4]. The most important known prognostic determinants for mortality are decline in lung function, acute exacerbations as well as pulmonary hypertension [5, 6].

Changes in the knowledge on etiology and pathogenesis of IPF, technical improvements in radiological diagnostics, as well as new treatment modalities resulted in a modification of the existing ATS/ERS/JRS/ALAT guidelines in 2011 [7]. Still, despite extensive research over the past 25 years, only two drugs have yet been identified as being effective in IPF, pirfenidone and nintedanib [8–10]. Hence, IPF remains a disease with a great unmet medical need [1, 11].

As the natural course of IPF is quite heterogeneous, and as the response to the novel anti-fibrotic drugs has been reported to show great variability, it is essential to identify reliable predictive factors indicating the risk of deterioration and the response to medical treatment, as well as side effects in a broad non-selected patient cohort [12, 13]. In addition, data collected in the frame of the numerous controlled clinical trials undertaken so far in IPF, here especially in the placebo arms, have provided important insights into the clinical course of IPF. It should, however, be kept in mind that patients recruited into these studies represent a rigorously selected population and do therefore not necessarily reflect the characteristics of IPF subjects seen in clinical routine.

In order to meet these demands, to better explore the pathogenesis and natural course of IPF, and also in order to facilitate translational research in biomaterials from IPF subjects, the European IPF Registry (eurIPFreg) and the European IPF Biobank (eurIPFbank) were set up in the frame of the European IPF Network, a research consortium funded by the European Commission under the FP7 program from 2008 until 2011 (see www.pulmonary-fibrosis.net). The eurIPFreg and eurIPFbank were launched in November 2009 to collect a broad range of clinical data and of biomaterials from European IPF patients in a longitudinal fashion [14]. Patients with other interstitial lung diseases and also other lung diseases were also included in the registry as comparator and disease control groups. EurIPFreg and EurIPFbank are comprised of the same cohort of patients; in this study only clinical registry data would be presented.

This manuscript describes baseline data of 525 recruited IPF subjects, covering the period from November 2009 until October 2016. It provides important insights into baseline characteristics, survival and management changes in European IPF patients over time.

## Methods

### Data collection

The eurIPFreg has been designed as an Internet-based, multicentre registry interlinked with the European IPF Biobank (eurIPFbank, see also www.pulmonary-fibrosis.net) [15]. The data protection concept was reviewed and approved by local and national networks such as the TMF (Technology*, Methods, and Infrastructure for Networked Medical Research e.V.)* and authorities (e.g. Hessian Data Protection Officer, Protocol Nr. 412,101 from 25.08.2008). Both, eurIPFreg and eurIPFbank have also been reviewed and received positive votes from institutional review boards in Germany (e.g. Ethics Committee of Justus-Liebig-University of Giessen; 111/08), France, Italy, Austria, Spain, Czech Republic, Hungary and the UK. The research was conducted strictly according to the principles of the Declaration of Helsinki. Patients were included into the registry starting November 2009. The eurIPFreg and eurIPFbank are listed in ClinicalTrials.gov (NCT02951416).

In the period between 2009 and 2016, the following sites recruited patients: Universities of Giessen and Marburg Lung Center, Germany and the nearby AGAPLESION Lung Clinic Waldhof-Elgershausen; Competence Center for Rare Pulmonary Diseases of Hopital Bichat in Paris, France; Interstitial Lung Disease Unit of Royal Brompton Hospital in London, United Kingdom; Reference Center for Rare Pulmonary Diseases, Centre Hospitalier Universitaire Dijon-Bourgogne, France; Dept. of Clinical and Molecular Biomedicine of Università degli Studi di Catania, Italy; Vienna University Hospital, Austria; Hospital Clinico San Carlos in Madrid, Spain; Department of Pulmonology Semmelweis University in Budapest, Hungary; Thomayer Hospital in Prague, Czech Republic; Ospedale San Gerardo in Monza, Italy; Università degli Studi di Napoli Federico II, Italy, and Ruhrlandklinik, University Hospital of Essen, Germany.

The software solution underlying the registry (secuTrial®) was developed by the German Parkinson Network and is certified by the Food and Drug Administration (FDA) as well as the European Medicines Agency (EMEA). The patients’ data are transferred via a secure internet-based data collection form and all data entries are based on the unique patient’s encrypted ID number (“pseudonym”).

The patients were eligible for the enrolment if they were at least 18 years old, had IPF (prevalent or incident cases) or other ILDs (as comparator group) as diagnosed by the expert site, and had provided written informed consent prior to the inclusion. In order to facilitate research, inclusion of other lung diseases such as lung cancer, COPD as well as healthy subjects or family members as additional comparator groups was made possible in the frame of an amendment in March 2013.

On a local level, each patient’s IPF diagnosis was evaluated in a multidisciplinary discussion including at least chest physicians, pathologists and radiologists on the basis of the respective ATS/ERS/JRS/ALAT guidelines. The registry had no explicit exclusion criteria, thereby reducing selection bias. The clinical data were collected at the time of enrolment (baseline) and in intervals 3 to 12 month thereafter (dictated by clinical routine).

After entry into the registry, each case was checked by a documentation officer (BP) for data quality and by FD and AG for internal plausibility of medical data and the diagnosis of IPF (e.g. hints for collagen/vascular diseases or hypersensitivity pneumonia in patient questionnaires). Also, in 2015 and 2016, all previous IPF cases diagnosed as having IPF according to the previous guideline from 2000, were checked if they would also fulfil the guideline criteria from 2011 [16]. No central reading of HRCT or histology samples was performed.

The clinical data acquisition took place primarily via patient and physician baseline questionnaires, which can be retrieved upon logging in to our website (www.pulmonary-fibrosis.net). The patient questionnaire included patient’s demographics, a detailed medical history making use of the WHO classification, complaints as well as report of co-morbidities [6]. Alongside with this questionnaire, the patient also received quality of life questionnaires, among them the EQ-5D, the SF-36 and the Mahler Index questionnaire [17–19]. These documents were available in different languages and were printed out on e-paper format and with an individual pseudonym on each page, allowing scanning and automated computer entry upon manual fill-out of the form.

The physician questionnaire contained data of physical examination and laboratory tests, pulmonary function, radiology, echocardiography, 6 MWD as well as other information concerning relevant patient’s diagnosis and therapy; the form had to be filled out online in English language. Import of historical medical data was also possible. Medication was assessed making use of the official WHO list of drugs 2018 from WHO Collaborating Centre for Drug Statistics and Methodology, allowing categorial (group-wise) analysis of co-medication (www.whocc.no/atc_ddd_index).

Next to the baseline data, follow-up data were obtained in a similar way, making use again of patient and physician questionnaires. These questionnaires consisted of a smaller number of items of the baseline questionnaires, but also included additional aspects relevant for the further course of the disease such as information on intermittent respiratory infections, working status, transplantation or any changes in the medication. In case a patient was deceased, the site investigator was asked to document this in the registry, including the underlying reasons of death (if known). In addition to the data as provided by the questionnaires, high resolution computed tomography (HRCT) and any other DICOM formatted images could be uploaded; during this procedure, the patient’s personal data in the DICOM header were replaced by the pseudonym.

Biological materials such as blood, bronchoalveolar lavage fluid (BALF) and tissue samples as well as exhaled breath condensates and electronic Nose (eNose) profiles were centrally recorded and managed through generation of patient-, time-, and specimen-specific Lab IDs and they were stored both, locally as well as in the centralized European IPF Biobank (eurIPFbank) located in Giessen.

### Quality of data and statistical analysis

Quality of data was improved by introduction of internal plausibility checks, in which different items were put into a logical context, causing the generation of queries in case inconsistent entries were noted (e.g. if physician’s and patient’s report were not consistent with regard to signs of underlying collagen/vascular disease). These queries were addressed to the respective site investigator, asking for clarification of the issue. In addition, site investigators were asked to conduct on-site data verification.

All statistical procedures were performed using SPSS 24 (SPSS, IBM Corp). For baseline data, the summary descriptive statistic was generated with categorical data displayed as absolute numbers and relative frequencies. Continuous data are shown as mean (SD) for normally distributed data or as median (interquartile range) for nonparametric data. Comparisons between groups were performed using a t-test or Mann–Whitney U test, as appropriate. For assessment of overall survival Kaplan-Meier analyses were applied.

## Results

The period of recruitment covered in this paper was chosen as November 2009–October 2016. By October 2016, a total of 2090 patients were included in eurIPFreg. One thousand eighty-six of these patients had suspected or proven ILD, among them 525 patients with IPF. The data of another 50 IPF patients, who had been recruited into the registry, could not be taken into consideration, as they were still under final assessment.

### Demographics

#### Patients with interstitial lung diseases other than IPF

**Fig. 1:**
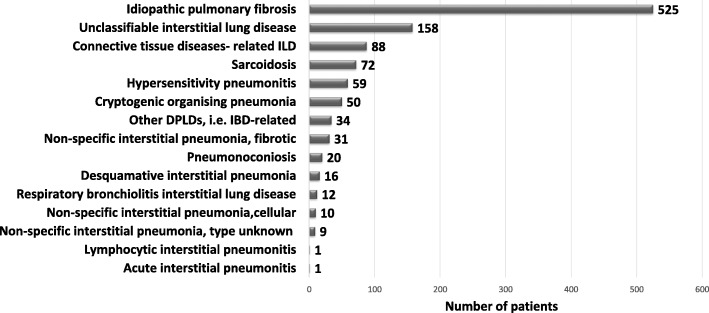
Distribution and diversity of ILD diagnoses in the eurIPFreg cohort. Data are presented as patients numbers per diagnosis. IBD: inflammatory bowel diseases; DPLD: diffuse parenchymal lung diseases

In the period between 2009 and 2016, a total of 1086 patients with ILDs were recruited into the eurIPFreg. The distribution of these ILDs is shown in Fig. 1: next to the 525 patients with IPF, there were 561 patients with other ILDs, who had a mean age of 65.2 ± 12.9 years, of them being 63.7% male. The most common clinical symptoms in these 561 non-IPF ILD cases were insidiously increasing dyspnea (85.9%), dry cough (50.4%) as well as fatigue (70.6%). A smoking history was reported by 64.7% of participants and 6.3% continued to smoke at the time of the enrolment. Despite profound evaluation, 158 patients (14% of all recruited patients) were found to be unclassifiable after multidisciplinary discussion (MDD).

#### Patients with IPF

With regard to the 525 IPF patients included in the eurIPFreg, the baseline characteristics are outlined in Table 1. Of all IPF patients, 18.64% had a familial history of IPF or other DPLD (Grades A-C).

**Table 1:** Clinical baseline characteristics of the IPF cohort

Demographic parameters	Value
Ethnical origin (% of the whole IPF cohort)	
Caucasian	67.6
African	3.8
Indian	1.3
Polynesian	1.0
Missing data (Patients did not provide the ethnic origin)	26
Male (%)	73.7
BMI	
mean value ± SD (kg-m^2^)	27.2 ± 4.6
Familial IPF (% of the whole IPF cohort)	
Grade A - Direct relative suffers or died at IPF / NSIP	9.5
Grade B - Direct relative suffers or died from IIP	4.4
Grade C - Direct relative suffers / died from non-classified lung disease	4.8
Age at diagnosis	
mean value ± SD (years)	65.2 ± 11.6
Age at enrollment into the registry	
mean value ± SD (years)	68.1 ± 11.1
Time between onset of symptoms and inclusion into eurIPFreg	
median; q1-q3 (months)	36.5; 19.2–70.3
mean value ± SD (months)	59 ± 3.85
Time between onset of symptoms and diagnosis	
median; q1-q3 (months)	6; 1–25.5
mean value ± SD (months)	21.8 ± 3.49
Smokers/ Ex-Smokers/ Never-Smokers (%)	4.0/65.4/30.6

**Fig. 2:**
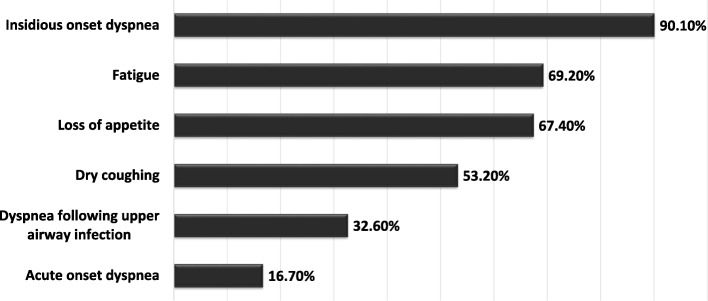
Distribution of self-reported symptoms of IPF patients. Data are presented as percentage of all patients with reported symptom

The most common clinical symptoms reported by the patients are shown in Fig. 2 and included insidious dyspnea, dry cough and fatigue. The mean time between self-reported onset of symptoms and IPF diagnosis was 21.8 months. The most common findings during the physical examination were crackles (95.5%), finger clubbing (30.8%), as well as pretibial oedema (9.1%).

To classify dyspnea in a patient questionnaire, we graded severity of dyspnea and impairment of physical activity in analogy to well-known NYHA classification [20]. Our cohort showed following distribution of severity of dyspnea in the IPF cohort upon enrolment in the registry: Grade I 12.6%, Grade II 45%, Grade III 33.6%, and Grade IV 8.8%.

### Diagnosis of IPF

Diagnoses of IPF were made based on the respective ATS/ERS/JPS/ALAT guidelines 2000 and 2011 [6, 7, 16]. In our study cohort, 151 patients were diagnosed according to the ATS/ERS Consensus Statement/2000, and 351 patients were diagnosed using criteria of ATS/ERS/JPS/ALAT guidelines released in 2011. Based on a retrospective review by AG and FD, the IPF patients diagnosed according to the guidelines 2000 also fulfilled the 2011 criteria.

### HRCT in IPF patients

Two different scales were used for the grading of HRCT. Prior to the release of the 2011 guidelines, the scale used in the eurIPFreg consisted of four grades and all the 151 patients diagnosed according to the 2000 guidelines were, independent of the existence of an open lung biopsy, classified into highly probable UIP (> 90% probability; 72.6% of patients), somewhat probable (75–90% probability; 20.5% of all patients), weakly probable (50–75% probability; 4.8%) and not probable UIP pattern (< 50% probability; 2.1% of all patients).

After the publication of the 2011 guidelines, the new HRCT classification (definite UIP pattern, possible UIP pattern and pattern inconsistent with UIP) was applied. In this regard, a definite UIP pattern was encountered in 63.7% of all IPF cases, a possible UIP pattern in 27.7%. In 8.6% of the cases an “inconsistent with UIP” pattern was found. In this cohort covering the time span between 2003 and 2014, 79% of the patients received open lung surgery incl. VATS; 3.5% did show features of UIP in coincidentally larger transbronchial biopsies, for which reason VATS was not recommended anymore. In all these cases, histological findings were consistent with a pathologic UIP pattern. In 17.5% of patients a surgical procedure was not recommended because of the general condition of the patients, but longitudinal follow up and discussion in a MDD rounds was very suggestive of UIP/IPF, for which reason this diagnosis was the preferred differential diagnosis.

### Bronchoscopy and lung biopsy in IPF

Data regarding bronchoscopy were available for 455 IPF patients (86.7%). According to these, bronchoscopy was performed as part of the diagnostic procedure in 309 out of these 455 patients (67.9%) and biopsies (incl. cryobiopsies) were taken in 128 cases, corresponding to 24% of all IPF patients.

BALF was performed in 263 cases (85.1%). The BAL differential revealed elevated neutrophil (14 ± 15.7%) and eosinophil (5.4 ± 8.5%) counts in face of normal lymphocyte (9.8 ± 10.7%) and reduced macrophage counts (71.2 ± 20.1% of all cells).

**Fig. 3:**
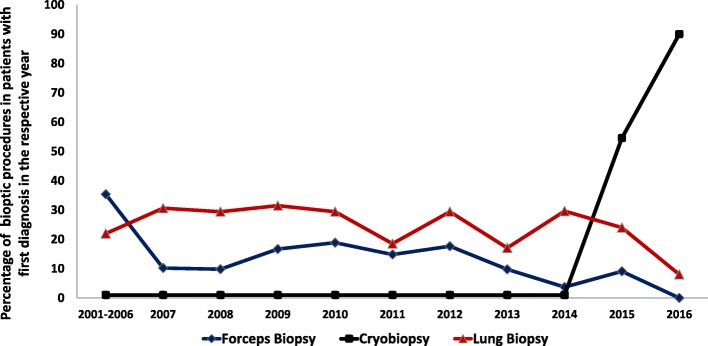
Change in biopsy procedures in IPF over time. Data are given as percentage of the respective procedure undertaken in IPF subjects in the year of first diagnosis

20–30% of all patients diagnosed as having IPF in 2010 or 2011 underwent open lung surgery or video-assisted thoracoscopy (VATS), but these numbers went down in following years, possibly due to the increasing use of cryobiopsy, as shown in Fig. 3.

When evaluating the whole DPLD cohort for the entire observation period (1086 patients from 2009 until 2016), we found that a pattern “inconsistent with UIP” was documented in 177 cases. Next to VATS, cryobiopsy was undertaken 19 (10.7%) of these cases and forwarded criteria of an UIP pattern (*n* = 12; 10 of which revealed fibroblast foci, spatial and temporal heterogeneity and absence of another pattern, hence only missing information as to the reference to the pleural surface), NSIP (*n* = 5), HP (n = 1), and unclassifiable IIP (n = 1). In addition, while we did not observe any exacerbation with a temporal association to the conductance of cryobiopsy, we have seen two exacerbations related to VATS.

### Baseline lung function in IPF patients

Baseline lung function data are displayed in Table 2. The mean FVC at the time of inclusion in the eurIPFreg was 2.39 ± 0.87 l, corresponding to 68.4% ± 22.6% of predicted value. One hundred thirty-five patients of the entire IPF cohort received long term oxygen treatment (LTOT), with a median flow of 2 l/min (range of 1-10 l/min). Of these 135 patients, only 18 received flow rates above 4 l/min. Six patients received LTOT with flow rates 8-10 l/min. Fifteen patients received non-invasive ventilation (BiPAP), with a mean EPAP of 7.5 ± 1.78 cmH20 and a mean IPAP of 17.6 ± 2.7 cmH20.

**Table 2:** Results of lung function and gas exchange data in the IPF cohort

Parameters	Mean value ± SD
VC (% predicted)	69.1 ± 21.5
FVC (% predicted)	68.4 ± 22.6
FEV 1 (% predicted value)	74.1 ± 31.7
FEV 1% FVC (% predicted)	110.2 ± 4.6
RV (% predicted)	74.2 ± 42.0
TLC (% predicted)	70.0 ± 38.4
DLCO (% predicted)	42.1 ± 17.8
pO2 (mm Hg) at rest	60.2 ± 20.1
pCO2 (mm Hg) at rest	37.7 ± 10.6

*Abbreviations*: *FEV1* Forced expiratory volume, *RV* Residual volume, *TLC* Total lung capacity, *VC* Vital capacity, *FVC* Forced vital capacity, *DLCO* diffusing capacity of the lung for carbon monoxide, *pO2* partial pressure of oxygen, *pCO2* partial pressure of carbon dioxide

### Echocardiography and six-minute walk test in IPF patients

Results of baseline echocardiography were available for 362 patients. An enlargement of the right heart was reported in 20.4% of the cases; an enlargement of the left ventricle or the left atrium was encountered in 12.9% of the patients. Signs of pulmonary hypertension were found in 16.8% of the patients, with systolic pulmonary arterial pressure (sPAP) values exceeding 50 mmHg (64 mmHg ± 18.9; mean ± SD). Tricuspid annular plane systolic excursion (TAPSE) of less than 1.5 cm was found in 14 patients (3.8%). The n-pro brain natriuretic peptide (nBNP)-value was assessed in 273 patients; the mean n-pro BNP was 301.24 pg/ml (range < 1–6716 pg/ml). Forty-one IPF patients (15%) showed an n-pro-BNP value exceeding 150 pg/ml.

A six-minute walk test was performed and was available at baseline for 420 patients (80%). The mean distance walked was 388 ± 122 m.

### Co-morbidities in IPF

**Fig. 4:**
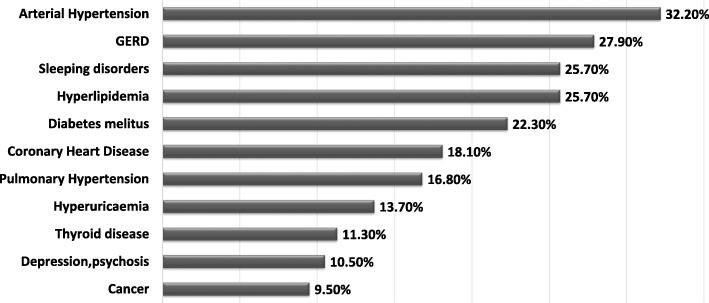
Spectrum of co-morbidities in the IPF cohort. Data are given as percentage of all patients. Multiple co-morbidities could be reported

Co-morbidities were common in patients with IPF and were assessed via both, physician- and patient questionnaires. Because co-morbidities are often exclusion criteria for clinical trials, this real-world data represents a broad IPF population without selection bias [21]. As shown in Fig. 4, the most common co-morbidity in our cohort was arterial hypertension, followed by gastro-oesophageal reflux.

### Treatment for IPF

**Fig. 5:**
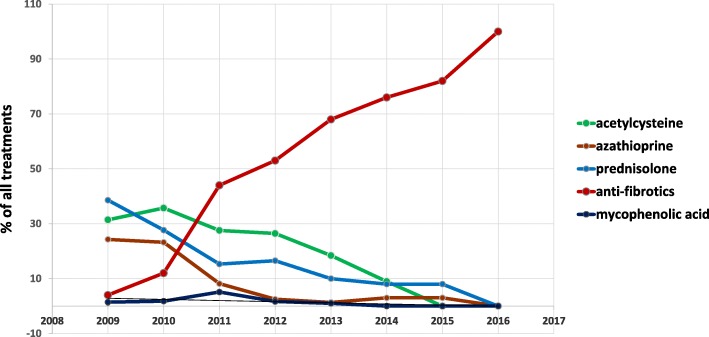
Change in IPF treatment over time. The graph shows various therapeutic regime (acetylcysteine, azathioprine, prednisolone, mycophenolic acid and anti-fibrotic drugs) in percentage of all treated patients

At the enrollment time, various therapeutic regimes were used throughout Europe. Prior to the commercial release of anti-fibrotic drugs pirfenidone and nintedanib, a significant number of the patients were treated with diverse immunosuppressants and/or N-acetylcysteine. In our study, the most used IPF medications were classified into following groups: antifibrotics (pirfenidone, nintedanib), N- acetylcysteine, prednisolone, azathioprine, as well as mycophenolic acid. Figure 5 displays the use of these therapy regimes in all participating European centers over time and in percentage of all treated patients, showing a quantitative replacement of any other therapy by the two antifibrotics drugs.

There is accumulating evidence that rehabilitation improves quality of life and symptoms in patients with IPF. In our cohort, 81 patients participated in an in-patient and 26 patients in an out-patient rehabilitation program.

### Outcomes of IPF patients

Of the 525 IPF patients included in the registry from November 2009 until baseline cut-off in October 2016, definite outcome data (date of death or last known visit) were available for 210 cases.

Of those, eight patients underwent lung transplantation (corresponding to 3.9%) and 78 patients (corresponding to 38%) had died until data cut-off in 2016. These 78 patients were enrolled in the registry at a mean age of 63.5 years, with a range of 42–88 years. The mean age of these subjects at the time of death was 71 years (range 44.5–90 years). The most common reasons for death were bronchopulmonary infections, i.e. pneumonia, leading to sepsis and multiorgan failure, followed by right heart failure due to progressive pulmonary hypertension.

**Fig. 6:**
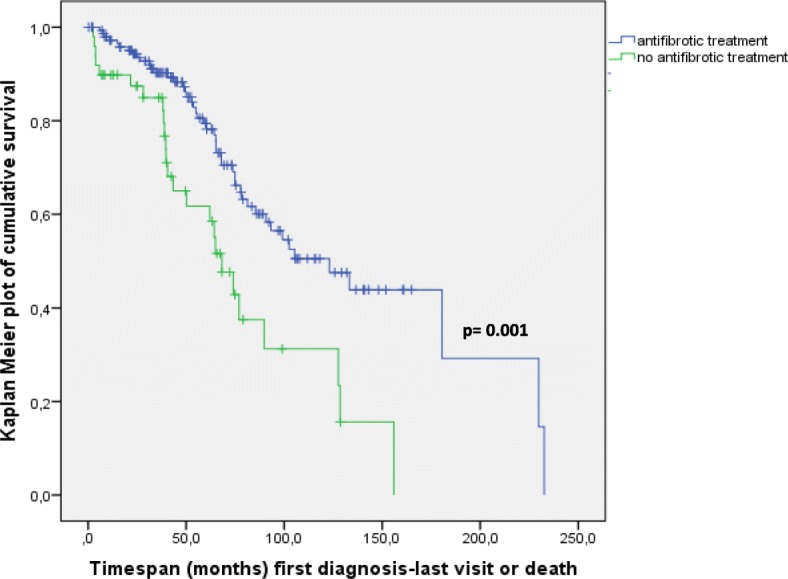
Overall survival of IPF patients upon first diagnosis depending on treatment. Given are Kaplan-Meier curves for cumulative survival, based on definite outcome data (survival status definitely known as per end of 2016) and on last visit data. A statistically significant difference in survival was encountered between patients receiving anti-fibrotic treatment and those not receiving antifibrotics, significance level p was 0.001. Within the group of patients receiving antifibrotic treatment, 83% of patients received pirfenidone and 17% received nintedanib

When assessing survival via Kaplan-Meier analysis in correlation to the date of first IPF diagnosis, our results indicate that the median survival on antifibrotics was 123.1 months (censored cases inclusive, range 84–162 months), as compared to a median survival of 68.3 months in patients treated with any other medication (censored cases inclusive, range 54–83 months). Figure 6 shows Kaplan-Meier analysis displaying improved survival in patients on anti-fibrotic medication vs. those receiving prednisolone or other treatment (*p* = 0.001).

The data reflect the inhomogeneity of the natural course of IPF in a “real world” clinical setting, ranging from slow progression with long periods of stable disease to a rapid progressive fibrosis, with successive lung function impairment and death within first 2 years upon diagnosis. In this study we did not explicitly compare outcomes of IPF vs. non-IPF cohort, leaving this topic to our further research.

## Discussion

In the frame of the European IPF Network “Natural course, Pathomechanisms and Novel Treatment Options in Idiopathic Pulmonary Fibrosis” (eurIPFnet), the multicenter, European-wide IPF registry (eurIPFreg) and biobank (eurIPFbank) were launched in 2009. In this report we summarize the clinical characteristics of our large European IPF cohort. Our study results include survival outcomes extend beyond baseline findings.

The primary purpose of eurIPFreg was to characterize a natural course of IPF, as well as to capture diagnostic and treatment strategies. This is in line with a previous note, according to which improved survival of IPF depends on better understanding of the epidemiology of the disease, its diagnostic spectrum and outcomes from emerging therapies [11, 22]. Furthermore, a real-life data registry significantly complements data from randomised controlled trials, typically comprising a wide range of severity, progression and co-morbidities.

With regard to demographics, the data obtained in our IPF cohort were similar to those reported in large randomized clinical trials and other registries [2, 23–29]. Pulmonary function tests in our cohort revealed a marked restrictive disease, with the FVC and DLCO being somewhat similar to the observations in the ASCEND study [30, 31].

Bronchoscopy was performed in a significant number of patients. Forceps biopsies, undertaken primarily in the first yeas of the patient recruitment, were reported to be somewhat indicative of interstitial lung disease in only about 25% of all the patients. As a result, VATS was a common procedure in the beginning of the registry, but significantly declined in numbers with the time due to knowledge of causing IPF exacerbations, but also with increasing use of less invasive cryobiopsy [32, 33]. BALF analysis of IPF patients showed increased numbers of neutrophils and eosinophils, in accordance with previous reports [7]. The mean distance in the 6 min walk test was 388 ± 122 m, which is similar to the data from other registries and clinical trials (420 m in AIPFR, 268 m in INSIGHTS-Reg., 415 m in ASCEND Trial) [2, 29].

The prevalence and impact of comorbidities on the clinical course of IPF remains unclear and is the subject of our further follow-up studies. From all reported co-morbidities, cardiovascular were most common, followed by GERD, sleep disorders, hyperlipidemia, diabetes and pulmonary hypertension. This appears to be also consistent with other registries [29, 34].

As the latest IPF guidelines were released in 2011, the eurIPFreg comprises patients initially diagnosed and treated according to the ATS/ERS Consensus Statement of 2000, as well as those diagnosed and treated according to the recent ATS/ERS/JRS/ALAT IPF guidelines of 2011 [7, 16]. Hence, the eurIPFreg is a useful tool in order to explore changes in the diagnostic and clinical practice in IPF in the last years. Likewise, pirfenidone and nintedanib have been commercially released in this time period.

In this regard, the diversity of this European IPF cohort may reflect not only the variety in natural course of the disease, but also changes in clinical management of the patients, especially when comparing our results to historic IPF cohorts [35]. Thus, the decline in the usage of prednisolone and other immunosuppressive medication reflects the implementation of recent IPF guidelines, and the knowledge arising from the PANTHER-IPF trial, according to which these therapeutic strategies are rather harmful than helpful in IPF [36, 37]. The use of prednisolone is nowadays restricted mostly to the therapy of severe exacerbations [38].

Our registry also reflects the steady increase of usage of antifibrotic drugs. In October 2016 for example 27 patients received nintedanib and 224 patients received pirfenidone. Our results showed an improved survival in patients on anti-fibrotic medication (*p* = 0.001), in accordance with randomized controlled trials, such as CAPACITY, ASCEND as well as open-label extension study (RECAP) [2, 39]. Prognostic indicators for IPF patients under antifibrotic treatment have yet to be established in the future, using epidemiological data and taking into account a larger numbers of patients with definite outcome data and longer follow up periods.

However, our data indicate that IPF still has a high mortality rate and that survival times are quite heterogenous. As mentioned above, these data may reflect both, the heterogeneous natural course of IPF in a clinical setting, ranging from stable disease to a rapid progressive fibrosis, and the change in the pharmacological approach to IPF subjects, with absence of antifibrotic drugs in the beginning, but widespread use at the end of the period.

The interpretation of the real-world data has several limitations. First, as a databank, the information available for each patient is based on clinical practice instead on a trial protocol. Despite our expectation to have the questionnaire for each visit and every patient fully completed, some categorical data were missing. Additionally, the co-medication, as well as co-morbidities, are partly self-reported as a part of the structured patient questionnaire and thus might lack accuracy.

## Conclusions

We report a clinical characteristic of a large European IPF cohort with outcome data extending up to 7 years. Our patients are diverse in age, impairment of lung function, therapeutic regimes and co-morbidities. The data reflect changes in the diagnostic and therapeutic approach in IPF in the last 10 years, supporting the important role of large real-world data registries to document and scrutinize changes in IPF management.

## Abbreviations

BALF: Bronchoalveolar lavage fluid
COPD: Chronic obstructive pulmonary disease
DLC0: Diffusing capacity of the lung for carbon monoxide
EMEA: European Medicines Agency
eNose: Electronic nose
eurIPFbank: European IPF Biobank
eurIPFreg: European IPF registry
FDA: Food and Drug Administration
FEV1: Forced expiratory volume
FVC: Forced vital capacity
hrCT: High resolution computed tomography
ILD: Interstitial lung diseases
IPF: Idiopathic pulmonary fibrosis
LTOT: Long term oxygen treatment
nBNP: n-pro brain natriuretic peptide
pCO2: Partial pressure of carbon dioxide
pO2: Partial pressure of oxygen
RV: Residual volume
sPAP: Systolic pulmonary arterial pressure
TLC: Total lung capacity
VC: Vital capacity

## References

[1] Martinez FJ (2017). The diagnosis of idiopathic pulmonary fibrosis: current and future approaches. Lancet Respir Med.

[2] Jo HE, et al. Baseline characteristics of idiopathic pulmonary fibrosis: analysis from the Australian idiopathic pulmonary fibrosis registry. Eur Respir J. 2017;49(2). 10.1183/13993003.01592-2016. 10.1183/13993003.01592-2016 28232409

[3] Hutchinson J (2016). Idiopathic pulmonary fibrosis: another step in understanding the burden of this disease. Eur Respir J.

[4] Lee HE (2016). Incidence and prevalence of idiopathic interstitial pneumonia and idiopathic pulmonary fibrosis in Korea. Int J Tuberc Lung Dis.

[5] Albera C (2016). Efficacy of pirfenidone in patients with idiopathic pulmonary fibrosis with more preserved lung function. Eur Respir J.

[6] Lynch DA, et al. Diagnostic criteria for idiopathic pulmonary fibrosis: a Fleischner society white paper. Lancet Respir Med. 2018;6(2):138–153 10.1016/S2213-2600(17)30433-2 10.1016/S2213-2600(17)30433-2 29154106

[7] Raghu G (2011). An official ATS/ERS/JRS/ALAT statement: idiopathic pulmonary fibrosis: evidence-based guidelines for diagnosis and management. Am J Respir Crit Care Med.

[8] Kolb M (2017). Nintedanib in patients with idiopathic pulmonary fibrosis and preserved lung volume. Thorax.

[9] Behr J (2017). German Guideline for Idiopathic Pulmonary Fibrosis - Update on Pharmacological Therapies 2017. Pneumologie.

[10] Rochwerg B (2016). Treatment of idiopathic pulmonary fibrosis: a network meta-analysis. BMC Med.

[11] Wilson JW, du Bois RM, King TE (2008). Challenges in pulmonary fibrosis: 8--the need for an international registry for idiopathic pulmonary fibrosis. Thorax.

[12] Behr J (2013). German guideline for diagnosis and management of idiopathic pulmonary fibrosis. Pneumologie.

[13] Loeh B (2015). Intraindividual response to treatment with pirfenidone in idiopathic pulmonary fibrosis. Am J Respir Crit Care Med.

[14] Guenther A (2008). International registry for idiopathic pulmonary fibrosis. Thorax.

[15] Guenther A, I.P.F.N. European (2011). The European IPF network: towards better care for a dreadful disease. Eur Respir J.

[16] American Thoracic Society (2000). Idiopathic pulmonary fibrosis: diagnosis and treatment. International consensus statement. American Thoracic Society (ATS), and the European Respiratory Society (ERS). Am J Respir Crit Care Med.

[17] Ley B (2012). A multidimensional index and staging system for idiopathic pulmonary fibrosis. Ann Intern Med.

[18] Kreuter M (2017). Health related quality of life in patients with idiopathic pulmonary fibrosis in clinical practice: insights-IPF registry. Respir Res.

[19] Pittrow D (2014). Symptom burden and health related quality of life in patients with idiopathic pulmonary fibrosis in clinical practice: insights-Ipf registry. Value Health.

[20] Genth S (1996). Comparison of NYHA classification with cardiopulmonary function in patients with chronic heart failure. Z Kardiol.

[21] Glaspole IN, et al. Determinants and outcomes of prolonged anxiety and depression in idiopathic pulmonary fibrosis. Eur Respir J. 2017;50(2). 10.1183/13993003.00168-2017. 10.1183/13993003.00168-2017 28818883

[22] Walsh SLF, et al. Diagnostic accuracy of a clinical diagnosis of idiopathic pulmonary fibrosis: an international case-cohort study. Eur Respir J. 2017;50(2). 10.1183/13993003.00936-2017. 10.1183/13993003.00936-2017 28860269

[23] Hutchinson J (2015). Global incidence and mortality of idiopathic pulmonary fibrosis: a systematic review. Eur Respir J.

[24] Agabiti N (2014). Idiopathic pulmonary fibrosis (IPF) incidence and prevalence in Italy. Sarcoidosis Vasc Diffuse Lung Dis.

[25] Esposito DB (2015). Idiopathic pulmonary fibrosis in United States automated claims. Incidence, prevalence, and algorithm validation. Am J Respir Crit Care Med.

[26] Raghu G (2014). Idiopathic pulmonary fibrosis in US Medicare beneficiaries aged 65 years and older: incidence, prevalence, and survival, 2001-11. Lancet Respir Med.

[27] Raghu G (2016). Incidence and prevalence of idiopathic pulmonary fibrosis in US adults 18-64 years old. Eur Respir J.

[28] Nalysnyk L (2012). Incidence and prevalence of idiopathic pulmonary fibrosis: review of the literature. Eur Respir Rev.

[29] Behr J (2015). Management of patients with idiopathic pulmonary fibrosis in clinical practice: the INSIGHTS-IPF registry. Eur Respir J.

[30] King TE (2014). A phase 3 trial of pirfenidone in patients with idiopathic pulmonary fibrosis. N Engl J Med.

[31] Richeldi L (2016). Nintedanib in patients with idiopathic pulmonary fibrosis: combined evidence from the TOMORROW and INPULSIS((R)) trials. Respir Med.

[32] Tomassetti S (2016). Bronchoscopic lung Cryobiopsy increases diagnostic confidence in the multidisciplinary diagnosis of idiopathic pulmonary fibrosis. Am J Respir Crit Care Med.

[33] Carbonelli C, Rossi G, Cavazza A (2015). Cryobiopsy and multidisciplinary diagnosis of idiopathic pulmonary fibrosis. Respirology.

[34] Oldham JM, Collard HR (2017). Comorbid conditions in idiopathic pulmonary fibrosis: recognition and management. Front Med (Lausanne).

[35] Martinez FJ (2005). The clinical course of patients with idiopathic pulmonary fibrosis. Ann Intern Med.

[36] Richeldi L, Abraham E (2007). Identifying patients with idiopathic pulmonary fibrosis: quality or quantity?. Am J Respir Crit Care Med.

[37] Idiopathic Pulmonary Fibrosis Clinical Research, N (2012). Prednisone, azathioprine, and N-acetylcysteine for pulmonary fibrosis. N Engl J Med.

[38] Behr J (2012). Prednisone, azathioprine, and N-acetylcysteine for pulmonary fibrosis. N Engl J Med.

[39] Fisher M (2017). Predicting life expectancy for Pirfenidone in idiopathic pulmonary fibrosis. J Manag Care Spec Pharm.

